# Assessment of the 10-Year Probability of Fracture Using Femoral Neck (FRAX) and Lumbar BMD (FRAXplus) in Menopausal Women with Non-Functioning Adrenal Tumors: Where We Stand Today (A Study-Focused Analysis)

**DOI:** 10.3390/jcm14072302

**Published:** 2025-03-27

**Authors:** Mihaela Stanciu, Oana-Claudia Sima, Mihai Costachescu, Ana Valea, Claudiu Nistor, Alexandra-Ioana Trandafir, Denisa Tanasescu, Tiberiu Vasile Ioan Nistor, Mihai-Lucian Ciobica, Mara Carsote

**Affiliations:** 1 Department of Endocrinology, “Lucian Blaga” University of Sibiu, Victoriei Blvd., 550024 Sibiu, Romania; mihaela.stanciu@ulbsibiu.ro; 2 Department of Endocrinology, Clinical County Emergency Hospital, 550245 Sibiu, Romania; 3 PhD Doctoral School of “Carol Davila” University of Medicine and Pharmacy, 010825 Bucharest, Romania; oana-claudia.sima@drd.umfcd.ro (O.-C.S.); mihai.costachescu@drd.umfcd.ro (M.C.); 4 Department of Radiology and Medical Imaging, Fundeni Clinical Institute, 022328 Bucharest, Romania; 5 Thoracic Surgery Department, “Dr. Carol Davila” Central Emergency University Military Hospital, 010825 Bucharest, Romania; 6 Department of Endocrinology, “Iuliu Hatieganu” University of Medicine and Pharmacy, 400012 Cluj-Napoca, Romania; 7 Department of Endocrinology, County Emergency Clinical Hospital, 400347 Cluj-Napoca, Romania; 8 Department 4—Cardio-Thoracic Pathology, Thoracic Surgery II Discipline, “Carol Davila” University of Medicine and Pharmacy, 0505474 Bucharest, Romania; 9 Medical Clinical Department, Faculty of Medicine, “Lucian Blaga” University of Sibiu, 550169 Sibiu, Romania; denisa.tanasescu@ulbsibiu.ro; 10 Medical Biochemistry Discipline, “Iuliu Hatieganu” University of Medicine and Pharmacy, 400347 Cluj-Napoca, Romania; tiberiu.nistor@umfcluj.ro; 11 Department of Internal Medicine and Gastroenterology, “Carol Davila” University of Medicine and Pharmacy, 020021 Bucharest, Romania; lucian.ciobica@umfcd.ro; 12 Department of Internal Medicine I and Rheumatology, “Dr. Carol Davila” Central Military University Emergency Hospital, 010825 Bucharest, Romania; 13 Department of Endocrinology, “Carol Davila” University of Medicine and Pharmacy, 020021 Bucharest, Romania; carsote_m@hotmail.com; 14 Department of Clinical Endocrinology V, C.I. Parhon National Institute of Endocrinology, 020021 Bucharest, Romania

**Keywords:** FRAXplus, FRAX, osteoporosis, fracture risk, surgery, menopause, adrenal, bone mineral density, DXA, cortisol

## Abstract

**Background/Objective:** Osteoporotic fractures may be prevalent, as expected, in patients with primary osteoporosis such as menopause-related or age-related bone loss, but a supplementary contribution to the risk may be added by less than common conditions, including a non-functioning adrenal tumor with or without mild autonomous cortisol secretion (MACS). Many of the standard fracture risk-related elements are captured by the FRAX model; yet, novel insights are brought by an improved algorithm, namely, FRAXplus. Our objective was to analyze the fracture risk in menopausal females diagnosed with low bone mineral density (BMD) and MACS-negative adrenal incidentalomas using FRAXplus (lumbar BMD adjustment). **Methods:** This as a retrospective, multi-center study of 66 menopausal women, where 50% of them had non-MACS adrenal tumors (group A), and 33 were controls (group B). They were put into four sub-groups, either group A1 (N = 14/33 subjects with normal DXA), or A2 (19/33 subjects with lowest T-score < −1), or group B1 (14/33) where subjects had normal DXA, or group B2 (19/33) for subjects with low BMD. **Results:** The sub-groups were matched on age, body mass index, and years since menopause, as well BMD matched (A versus B, A1 versus B1, A2 versus B2). FRAX analysis showed similar results for 10-year probability between groups A and B, and A2 and B2, while lumbar BMD adjustment showed statistically significant lower risk in group A1 versus B1 (*p* = 0.013), but not for hip fracture (*p* = 0.064). **Conclusions:** we introduced a pilot study in the FRAXplus model regarding adrenal tumors diagnosed in menopausal females with or without low BMD at central DXA assessment, a pilot study that to the best of our knowledge represents the first of this kind due to the novelty of using this fracture risk calculator with lumbar BMD adjustment. FRAXplus algorithm might be a better discriminator for fracture risk in these patients since we found that in age-, BMI-, and years since menopause-matched sub-groups, patients with normal DXA and MACS-free adrenal incidentalomas display a lower 10-year probability of major osteoporotic fractures than controls upon lumbar BMD adjustment.

## 1. Introduction

Fragility fractures are mostly prevalent, as expected, in patients with primary osteoporosis or osteopenia according to the menopause- or age-related bone loss, but a supplementary contribution to the fracture risk in these subjects may be added by less common conditions, including certain drugs (other than glucocorticoids), autoimmune ailments, or endocrine tumors [1,2,3]. For instance, MACS (mild autonomous cortisol secretion) in adrenal incidentalomas brings a heterogeneous risk of deteriorated skeletal health which is currently less understood, as opposite to the one found in subjects with a full-blown clinical picture and landscape of complications in Cushing’s syndrome of either pituitary or adrenal cause [4,5,6]. MACS-adrenal tumors represent one third of the general category involving non-functioning adrenal tumors, with an age-dependent increasing incidence varying from 1–5% up to 10–15% in certain sub-groups [7,8,9]. The autonomous cortisol excess might have multiple negative effects (e.g., cardio-metabolic or osseous) despite the fact that many individuals are apparently healthy, considering that the identification of the adrenal mass occurred accidentally via performing an abdominal ultrasound, computed tomography, or magnetic resonance imaging for unrelated purposes [10,11,12]. Notably, the higher rate of co-morbidities in menopausal women or seniors diagnosed with adrenal incidentalomas has been found by some authors in all patients with a non-functioning adrenal tumor, regardless of MACS [13,14,15].

The tools to estimate the 10-year probability of fractures have been developed to assist clinicians in everyday practice with detecting the high-risk individuals who should undergo a further specific evaluation of their bone status and then promptly start medication against osteoporosis [16,17,18,19]. Many (but not all) of the fracture risk-related contributors are already captured by the conventional FRAX model; yet, novel insights are brought by an improved algorithm version of the current model, namely, FRAXplus, which is currently under beta testing and it is expected to offer a wider perspective of the risk evaluation via introducing new inputs such as lumbar bone mineral density (BMD), the history of fall, or type 2 diabetes duration [16,17,18,20,21]. Of note, while the conventional algorithm allows for a risk estimation by using or not using results regarding the femoral neck bone mineral density (BMD) according to a central DXA (dual-energy X-ray absorptiometry) scan, many individuals, including those diagnosed with MACS-positive/negative adrenal incidentalomas, might display the lumbar BMD as the most affected DXA site. Hence, a fracture risk underestimation might be registered. The estimation using lumbar BMD has been recently added to the model (FRAXplus) [18].

### Objective

We aimed to analyze the fracture risk in menopausal females diagnosed with low BMD from a central DXA (non-normal results) and with MACS-negative adrenal incidentalomas under the FRAXplus model perspective, which, to the best of our knowledge, represents the first study of its kind.

## 2. Material and Methods

### 2.1. Study Design

This was a retrospective, multi-center, single-country, cross-sectional, pilot study in menopausal women who had a central DXA scan between January 2023 and January 2024.

### 2.2. Studied Population

The studied group (A) included menopausal patients confirmed with MACS-negative adrenal incidentalomas, and group B included menopausal subjects without any adrenal tumors (or controls). The designation of sub-group A1/A2 or B1/B2 came from the BMD results from a central DXA, meaning A1 or B1 groups included subjects with normal DXA results (lowest T-score at central DXA between −1 and +1 SD), respectively, and A2 or B2 included those with non-normal DXA results (osteopenia or osteoporosis, meaning the individuals had the lowest T-score at central DXA of <−1 SD). Both groups had an abdominal computed tomography scan, and group A included patients who were found with an adrenal incidentaloma, while control group (group B) was found negative for any suspected or confirmed adrenal masses.

Inclusion criteria were menopausal status, informed written signed consent, and criteria for MACS-free (non-functional) adrenal incidentalomas (for group A), meaning a second-day plasma cortisol of less than 1.8 µg/dL following a 1 mg-dexamethasone overnight suppression test. Exclusion criteria were active cancers; bone metabolic and genetic diseases; primary hyperparathyroidism; full-blown (clinically manifested) Cushing’s syndrome; acromegaly; Conn’s disease; pheochromocytoma; history of unilateral or bilateral adrenalectomy; acute infections; pediatric population; non-usable data from a central DXA; prior or current glucocorticoid exposure; lack of abdominal computed tomography scan or results; prior specific medication against osteoporosis or previous diagnosis of osteoporosis/osteopenia.

### 2.3. Study Protocol

Data regarding age and years since menopause were collected for each patient and body mass index (BMI) was calculated. Computed tomography scans were performed in each center for unrelated purposes to the adrenal tumors (e.g., trauma, kidney stones, etc.). The females who were detected with adrenal incidentalomas were pre-selected for group A and the others became control group (group B).

Further on, the patients who were pre-selected amid the imaging scan (that identified an adrenal incidentaloma) further underwent hormonal testing. The patients with pheochromocytoma or Conn’s syndrome confirmation were excluded, as mentioned. The exploration of the pituitary–adrenal axes with 1 mg dexamethasone suppression testing excluded the patients who displayed a higher second-day plasma cortisol than 1.8 µg/dL [blood samples were required, measuring: baseline ACTH (adrenocorticotropic hormone), baseline serum morning cortisol, and serum cortisol after 1 mg dexamethasone administration at 11 p.m.].

**Figure 1:**
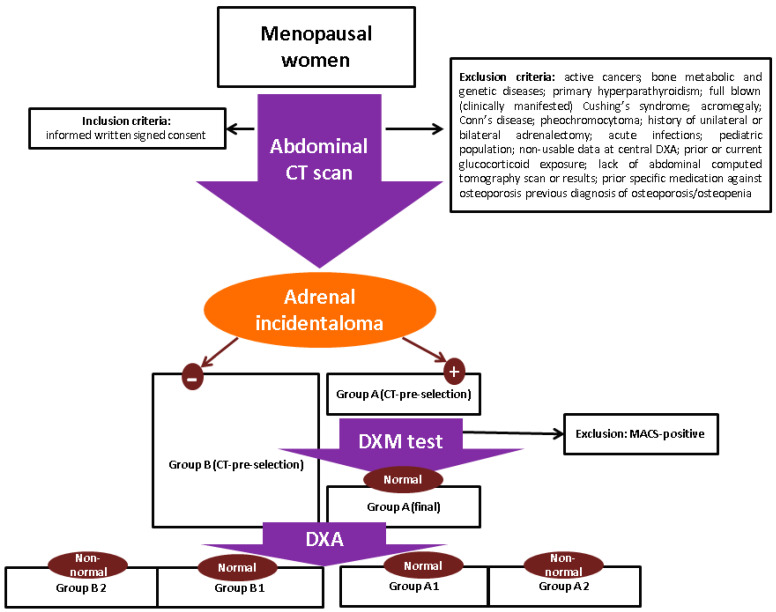
Flowchart diagram of the study protocol.

The individuals that were pre-selected based on CT scan and normal dexamethasone testing were then checked for their DXA results. Bone status was assessed by a central DXA using a GE Lunar device (at lumbar spine, femoral neck, and total hip). The DXA provided BMD and an associated T-score for each site, and low bone mass was considered based on the lowest central T-score of <−1 regardless of the site [22,23] (Figure 1).

**Figure 2:**
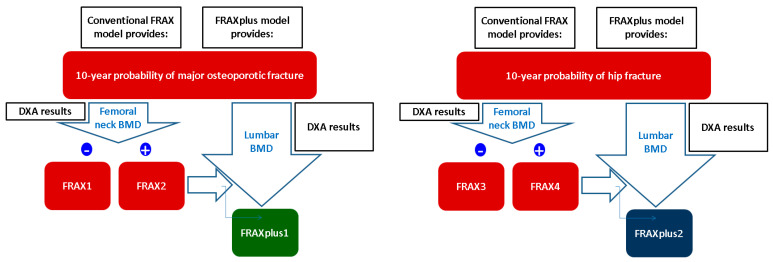
The estimation of 10-year probability of major osteoporotic fractures, respectively, and hip fracture (the terms according to the current study).

10-year probability of fracture was estimated using FRAX and FRAXplus models (adjusted for lumbar spine BMD) for each patient and the resulting scores were classified as follows (Table 1, Figure 2).

### 2.4. Statistical Analysis

The data were analyzed using Microsoft Excel 16.87 and IBM SPSS 29.0.2.0. Central tendencies were demonstrated using the mean ± standard deviation (SD) or as quartiles (Q1, median/Q2, Q3). Groups were compared using Student’s *t*-test or the Mann–Whitney test. Correlation coefficients were computed using Kendall’s tau method.

### 2.5. Ethical Considerations

Each subject signed an informed consent form during hospitalization in each hospital according to the local standard protocols. The Declaration of Helsinki was respected. The retrospective data analysis was approved by the local Ethical Committees (702-28 June 2024; 665-31 January 2024; 124-25 June 2024; 6284-8 February 2024; 2058-30 January 2024).

## 3. Results

### 3.1. Demographic Features

A total of 66 patients were included, 33 subjects were confirmed with non-MACS adrenal tumor (representing group A), and 33 controls (group B which included individuals without any adrenal masses). A total of 14 patients in group A had a normal DXA (group A1), and 19 had low BMD (group A2); 14 patients in group B had a normal DXA (group B1), and 19 had non-normal DXA results (group B2).

Mean age in group A, years since menopause, and BMI versus group B, are as follows (61.21 ± 10.94 versus 62.00 ± 6.23 years; 14.79 ± 10.78 versus 14.97 ± 6.60 years, respectively, 28.18 ± 5.34 versus 28.88 ± 3.56 kg/m^2^, *p* > 0.5 for each) (Table 2).

**Figure 3:**
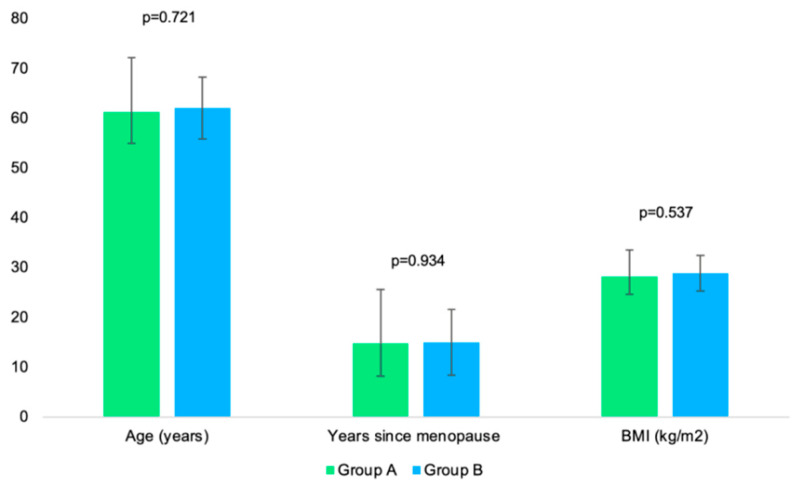
Bar charts showing the mean age, number of years since menopause, and BMI between group A and B (error bars showing the standard deviation).

No statistically significant differences regarding age, years since menopause, and BMI were observed between the mentioned sub-groups A1 and A2, A1 and B1, A2 and B2, or B1 and B2, respectively (Figure 3).

### 3.2. Hormonal Panel in Women with Adrenal Tumors

The analysis for group A showed a mean ACTH of 15.65 ± 13.70 pg/mL, of 19.10 ± 16.72 pg/mL in group A1, and of 12.45 ± 9.81 pg/mL in group A2. Mean baseline serum cortisol was 13.35 ± 5.55 μg/dL in group A, 13.75 ± 3.74 μg/dL in group A1, and 13.03 ± 6.78 μg/dL in group A2. The mean second-day plasma cortisol after a 1 mg dexamethasone suppression (low dose) test was 1.23 ± 0.36 μg/dL in group A, 1.13 ± 0.44 μg/dL in group A1, and 1.33 ± 0.27 μg/dL in group A2 (Table 3).

### 3.3. DXA Analysis Based on the Lowest T-Score in Each Patient at Central DXA Scan

The average lowest T-score at central DXA was similar between group A and B (*p* = 0.785), as well as between group A1 and B1 (*p* = 0.580), and between group A2 and B2 (*p* = 0.334) (Table 4).

### 3.4. DXA Analysis for Each Central DXA Site

**Figure 4:**
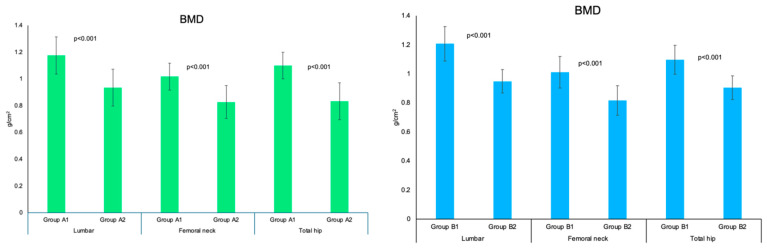
Bar charts showing lumbar, femoral neck, and total hip mean BMD in group A1 and group A2 (**left**), respectively, and B1 and B2 (**right**); error bars showing the standard deviation.

Lumbar, femoral neck, and total hip BMD/T-score were similar between groups A and B, as well as between groups A1 and B1, and A2 and B2. BMD/T-score for all mentioned sites were statistically significantly lower in group A2 versus A1, respectively, than group B2 versus B1 (*p* < 0.001) (Table 5 and Figure 4).

### 3.5. 10-Year Probability of Fracture: Conventional FRAX Model

The values of FRAX1, FRAX2, FRAX3, and FRAX4 were similar between group A and B, and between group A2 and B2. FRAX2 was statistically significantly lower in group A1 versus B1 (*p* = 0.030).

Group A1 had a statistically significant lower value of FRAX1 versus group A2, with a median (IQR) of 2.20 (1.40, 4.10) % versus 4.70 (3.35, 7.15) % (*p* = 0.05); of FRAX2 [1.85 (1.40, 3.10) % versus 4.35 (3.20, 6.20) % (*p* = 0.004)]; of FRAX3 (*p* = 0.037); and FRAX4 [0.15 (0.00, 0.20) % versus 0.75 (0.30, 1.90) %; *p* = 0.002)]. However, in the control group, only FRAX2 was statistically significantly lower in group B1 versus B2 (*p* = 0.037), and FRAX4 (*p* = 0.002) (Table 6).

### 3.6. 10-Year Probability of Fracture: Novel FRAXplus Model

FRAXplus analysis [18] between group A and B showed that they had similar FRAXplus1 [2.80 (1.55, 4.40) % versus 3 (2.25, 3.45) %; (*p* = 0.411)] and FRAXplus2 [0.30 (0.10, 1.10) % versus 0.30 (0.10, 0.65) %; (*p* = 0.707)] results. FRAXplus1 was statistically significantly lower in group A1 versus B1 (*p* = 0.013), but not FRAXplus2 (*p* = 0.064). Group A1 had a statistically significantly lower FRAXplus1 [1.55 (1.30, 2.50) %] compared to group A2 [3.30 (2.70, 4.60) %; *p* = 0.004], as well as FRAXplus2 [0.10 (0.00, 0.10) % versus 0.55 (0.30, 1.40) %; (*p* < 0.001)]. The control group showed only FRAXplus2 to be statistically significantly lower in group B1 versus B2 (*p* = 0.002) (Table 7).

### 3.7. 10-Year Probability of Fracture: Conventional Versus Novel Model of Risk Estimation

The differences between the estimation according to both models were analyzed. In group A: FRAXplus1 was statistically significantly lower compared to FRAX1 (*p* < 0.001), respectively, to FRAX2 (*p* < 0.001). FRAXplus2 was statistically significant lower versus FRAX3 (*p* = 0.023), and FRAX 4 (*p* < 0.001) (Table 8).

**Figure 5:**
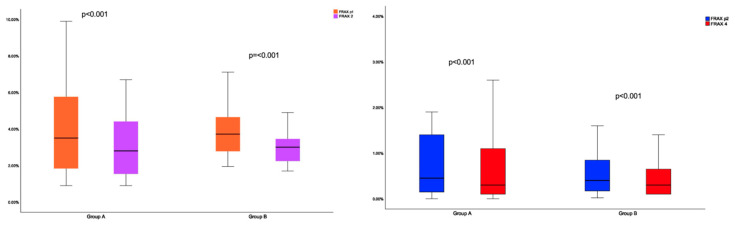
Boxplots showing the results for FRAXplus1 (orange box) versus FRAX2 (magenta box) between group A and group B (**left**); FRAXplus2 (blue box) versus FRAX4 (red box) between group A and group B (**right**).

In group B, FRAXplus1 was statistically significantly lower compare to FRAX1 and FRAX2 (*p* < 0.001), and FRAXplus2 was statistically significantly decreased versus FRAX3 (*p* = 0.004) and FRAX4 (*p* < 0.001) (Figure 5).

Group A1 showed a statistically significantly lower FRAXplus1 versus FRAX1 (*p* = 0.011) and FRAX2 (*p* = 0.008), as well as a lower FRAXplus2 versus FRAX3 (*p* = 0.011). Group A2 associated FRAXplus1 was lower versus FRAX1 and FRAX2 (*p* < 0.001 for each), while FRAXplus2 was similar with FRAX3 (*p* = 0.191), but statistically significantly decreased versus FRAX4 (*p* < 0.001).

**Figure 6:**
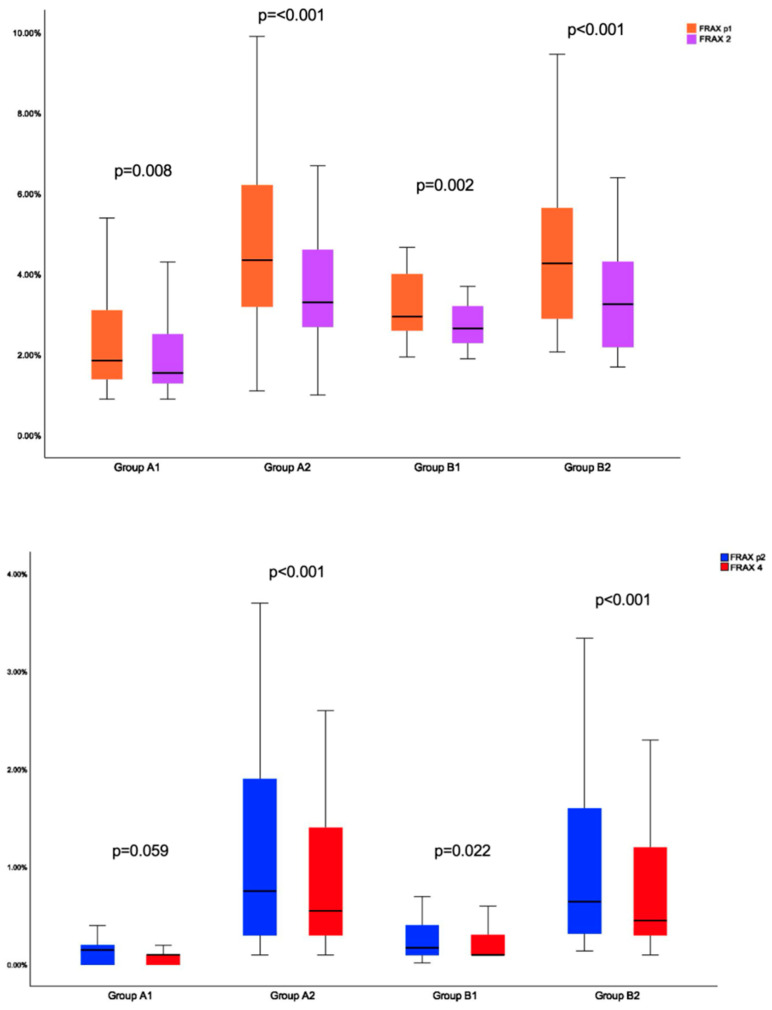
Boxplots showing the distribution of FRAXplus1 (orange) and FRAX2 (magenta) within groups A1 and A2, and B1 and B2 (upper capture); of FRAXplus2 (blue) and FRAX4 (red) within groups A1 and A2, and B1 and B2 (lower capture).

Group B1 had a statistically significantly decreased FRAXplus1 versus FRAX1 (*p* < 0.001) and FRAX2 (*p* = 0.002), and a lower FRAXplus2 compared to FRAX3 (*p* < 0.001) and FRAX4 (*p* = 0.022). Group B2 showed a FRAXplus1 lower than FRAX1 (*p* = 0.031) and FRAX2 (*p* < 0.001); FRAXplus2 was similar to FRAX3 (*p* = 0.483) but lower than FRAX4 (*p* < 0.001) (Figure 6).

### 3.8. 10-Year Probability of Fracture According to the Novel Model: Correlations with Years Since Menopause and Total Hip BMD

FRAXplus1 statistically significantly correlated with the age of the subjects within all studied groups (the lowest correlation coefficient being 0.590 in group B1, and the highest being 0.874 in group A1), while the years since menopause (which is not a FRAX/FRAXplus input) correlated with FRAXplus1, displaying statistically significant correlation coefficients (except for group B1) between 0.351 (in group B) and 0.730 (in group A1). On the other hand, total hip BMD showed statistically significant negative correlations with FRAXplus1 in group A (r = −0.563, *p* = 0.002), group B (r = −0.415, *p* < 0.001), and group B2 (r = −0.616, *p* < 0.001) (Table 9).

**Figure 7:**
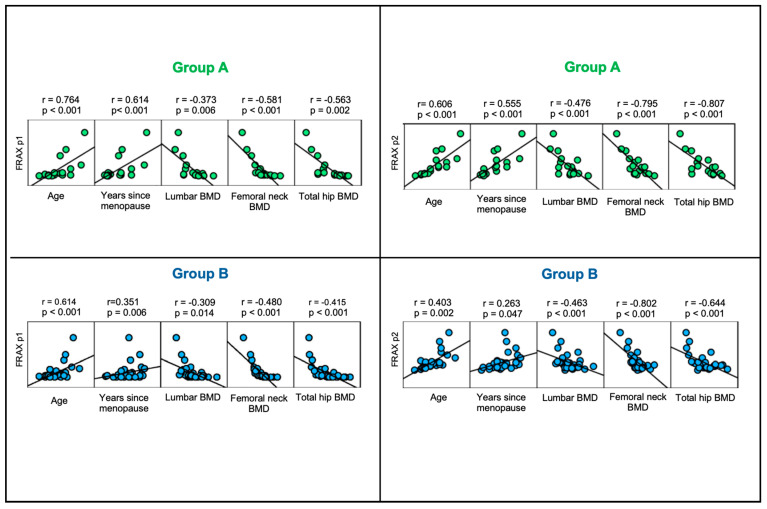
Scatterplot matrix showing the correlations between FRAXplus1 and FRAXplus2 with age, years since menopause, lumbar BMD, femoral neck BMD, and total hip BMD in group A and B.

FRAXplus2 statistically significantly correlated with the subjects’ age in the studied groups (except for group A1 and B1), displaying correlation coefficients between 0.403 (in group B) and 0.606 (in group A), and with years since menopause in groups A, A2, B, and B2 (correlation coefficients varied between 0.263 in group B and 0.555 in group A). Total hip BMD had a negative correlation with FRAXplus2 in all studied groups (statistically significant Kendall rank correlation coefficients varied between −0.430 in group B1 and −0.807 in group A (Figure 7)).

## 4. Discussion

In this pilot study, we introduced analysis beyond the conventional FRAX model (which is currently available all over the world) [16,17], specifically, the FRAXplus algorithm [18] (that is still under beta testing and it has not been widely released yet). We applied the risk calculation in menopausal women who were not on anti-osteoporotic medication with or without non-functional adrenal tumors (N = 66). The sub-groups had either a normal central DXA or non-normal one (osteopenia or osteoporosis), these results being newly diagnosed and/or confirmed amid this protocol of the retrospective data collection.

To the best of our knowledge, this study represents the first of this kind due to the novelty of using this type of fracture risk estimation with improved inputs such as lumbar BMD according to a central DXA assessment [18]. This approach seems particularly useful in patients who present incongruences in the level of BMD deterioration between the central DXA sites or subjects with less-described conditions in terms of associated fragility fracture risk as found in the field of adrenal tumors in menopausal women or even senior males who do not display clear features of Cushing’s syndrome [22,23,24].

The analyzed sub-groups showed age, years since menopause, and BMI matches, as well as BMD matches (group A versus B, A1 versus B1, and A2 versus B2), and this is particularly important in order to highlighting the differences that might be found when estimating the 10-year probability of fracture in the mentioned cohorts. While the estimated risk for major osteoporotic/hip fractures according to the conventional model mostly showed a lower value in the groups with higher BMD (non-normal DXA) versus the groups with a normal DXA, and a similar risk between the BMD-matched sub-groups (except for 10-year hip fracture risk which statistically significant decreased in A1 versus B1 group). The 10-year probability of major osteoporotic fractures as adjusted according to the use of lumbar BMD showed heterogeneous results: females with tumors versus controls (A versus B), and the sub-groups with a non-normal DXA (A2 versus B2) showed a similar fracture risk, while the sub-groups with normal BMD (A1 versus B1) displayed a lower risk in the tumor group than the control group which might prove that the current model overestimates the fracture risk as opposed to using lumbar BMD [18] for the risk estimation. We suggest that patients with a normal DXA and MACS-free adrenal incidentalomas display a lower 10-year probability of major osteoporotic fractures than controls upon lumbar BMD adjustment due to potential asymmetrical involvement of the bone mass between central DXA sites (as differently used according to the standard versus novel FRAX algorithm). Moreover, we might speculate another fact: the current classification of MACS/MACS-free tumors might not cover all hormonal aspects (particularly across a longitudinal perspective) and a mild hormonal activity may not be reflected by the current definition that only takes into consideration the value of the plasma cortisol following dexamethasone administration. For instance, some data suggests that a low-normal baseline ACTH as the single input, or in addition to the plasma, urinary, or salivary cortisol assays, might change the perspective of defining MACS/non-MACS incidentalomas. Further studies are necessary to highlight novel cutoffs and standard criteria [22,23,24].

Interestingly, this observation did not apply for the 10-year hip fracture probability (we found a borderline significance of *p*-value between A1 and B1 of 0.064). Moreover, the 10-year probability of hip fracture reflected a higher risk in those groups with a lower BMD (group A1 versus A2, *p* < 0.001; group B1 versus B2, *p* = 0.002). A rather decreased 10-year risk of any osteoporotic fracture in the entire cohort might not capture the true essence of using lumbar BMD for this estimation.

Currently, unless clear Cushing’s syndrome, the data on the traditional FRAX model with respect to the subjects confirmed with various adrenal masses are limited, and, generally, the bone status evaluation that has been performed in MACS-positive and MACS-free patients with adrenal incidentalomas has showed inconsistent results [25,26]. Overall, one-third of the non-functioning adrenal tumors (which are reported with a 5% up to 20% prevalence in adults) are MACS-positive, and two-thirds of the entire category is more affected by hypertension or type 2 diabetes than having a clear diagnosis of osteoporosis, despite a higher fracture risk than the general population, particularly, in menopausal women [27,28,29]. In addition, the presence of an osteoporosis/osteoporotic fracture in a menopausal woman with an adrenal incidentaloma does not necessarily indicate an adrenalectomy (unless other key findings are co-identified, such as an elevated cardiovascular and metabolic risk or increased size at serial imaging evaluation), yet, unexplained bone loss and/or incidental fractures during follow-up in such patients might raise the issue of surgery (independently of other indications for the adrenal removal) in order to prevent the fracture risk increasing [30,31,32]. Hence, bone health assessment might become an essential element in the tailored management of menopausal females with adrenal incidentalomas, and we need adequate algorithms to estimate the fracture risk in such patients in order to offer them an early interventional approach against osteoporosis and to facilitate fracture risk reduction. To date, the issue of MACS-positive tumors is not a matter with a distinct guideline with regard to the fracture risk cross-approach in terms of identification, stratification, and multimodal strategy regarding both the tumor and the bone.

Limits of the current study are the sample size of the cohort and the retrospective design. A total of 66 patients were included and 50% of them had adrenal tumors. Noting this is a pilot study using FRAXplus [18] which is not freely accessible at this point, a further expansion of these data is mandatory, including in prospective trials. Moreover, the studied population represented menopausal women who checked the complex list of exclusion criteria (in order to avoid a multidisciplinary panel of co-elements that might contribute to the bone loss and bring an additional bias [7,33,34,35]), and, hence, it limited the number of enrolled subjects. Also, paradoxically, since it is an FRAX/FRAXplus input, we found no statistically significant correlation between BMI and FRAXplus1 or FRAXplus2 in any of the studied sub-groups and some other co-factors might mislead the interpretation of these results such as the metabolic complications (e.g., type 2 diabetes mellitus and metabolic syndrome) in menopausal patients with adrenal incidentalomas [13,36,37,38,39].

As strengths of the study, we found that FRAXplus1 [18] was statistically significantly lower in group A1 versus B1 (*p* = 0.013), but not FRAXplus2 (*p* = 0.064), hence, pointing out the potential role for this type of adjustment to the standard FRAX model that has already been incorporated into practice guidelines amid these 15 years since its initial launch [16,17,18,19]. Also, group A1 had a statistically significantly lower FRAXplus1 (of 1.55%) compared to group A2 (of 3.30%; *p* = 0.004), as well as their FRAXplus2 being lower (of 0.10% versus 0.55%, *p* < 0.001). Larger trials will point out which fracture risk calculator is the most useful according to a personalized decision in each case, including menopausal women with non-functioning adrenal incidentalomas. Such algorithms might cover the heterogeneous landscape in relationship with less common ailments such as adrenal tumors (other than those manifested with classical Cushing’s syndrome features).

## 5. Conclusions

We introduced a pilot study in the FRAXplus model using menopausal females with adrenal tumors diagnosed with or without low BMD at a central DXA assessment. To the best of our knowledge, this pilot study represents the first of this kind due to the novelty of using this fracture risk calculator with lumbar BMD adjustment. The FRAXplus algorithm might be a better discriminator for fracture risk in these patients since we found that in age-, BMI-, and years since menopause-matched sub-groups, patients with a normal DXA and MACS-free adrenal incidentalomas display a lower 10-year probability of major osteoporotic fractures than controls upon lumbar BMD adjustment.

## Figures and Tables

**Table 1:** 10-year osteoporotic fracture risk assessment based on current FRAX model and FRAXplus model (we introduced the terms “FRAX”, alternatively “FRAXplus” + “number” to pinpoint the 10-year probability of fracture that has been applied amid the statistical analysis); * green font within the table means FRAXplus model for major osteoporotic fractures risk estimation; ** bluefont within the table means FRAXplus model for hip fracture risk estimation.

Designated Terms Amid the Current Study	10-Year Fracture Risk Assessment for:	Site of Bone Mineral Density (at Central DXA) That Has Been Used for the Risk Estimation:	FRAX Model
FRAX1	major osteoporotic fractures	none	conventional FRAX
FRAX2		with femoral neck BMD	
FRAXplus1 *		with femoral neck BMD + lumbar BMD	FRAXplus
FRAX3	hip fracture	none	conventional FRAX
FRAX4		with femoral neck BMD	
FRAXplus2 **		with femoral neck BMD + lumbar BMD	FRAXplus

**Table 2:** Analysis of age, years since menopause, and BMI in the studied groups [group A (menopausal patients confirmed with MACS-negative adrenal incidentalomas) and group B (menopausal subjects without any adrenal tumors or controls); the designation of A1/A2 or B1/B2 comes from the BMD results at central DXA, meaning A1 or B1 with normal DXA results, respectively, and A2 or B2 with non-normal DXA results (osteopenia or osteoporosis)].

Group	Age (Years) Mean ± SD	Years Since Menopause Mean ± Standard Deviation	Body Mass Index (kg/m^2^) Mean ± Standard Deviation
**A**	61.21 ± 10.94	14.79 ± 10.78	28.18 ± 5.34
**A1**	57.29 ± 10.49	10.93 ± 8.17	30.05 ± 4.73
**A2**	64.11 ± 10.61	17.63 ± 11.77	26.91 ± 5.47
**B**	62.00 ± 6.23	14.97 ± 6.60	28.88 ± 3.56
**B1**	61.50 ± 5.23	14.71 ± 6.98	28.29 ± 3.54
**B2**	62.37 ± 6.99	15.16 ± 6.50	29.32 ± 3.61
***p*-value between:**			
**Groups A and B**	0.721	0.934	0.537
**Groups A1 and A2**	0.077	0.077	0.103
**Groups A1 and B1**	0.194	0.199	0.141
**Groups A2 and B2**	0.555	0.429	0.118
**Groups B1 and B2**	0.699	0.852	0.420

**Table 3:** Mean ACTH, baseline serum cortisol, and overnight serum cortisol upon 1 mg overnight dexamethasone suppression test in group A, A1, and A2.

Group	Plasma ACTH (pg/mL) Mean ± SD	Morning Plasma Cortisol (μg/dL) Mean ± SD	Plasma Cortisol Second Day After 1 mg Dexamethasone Test (μg/dL) Mean ± SD
**A**	15.65 ± 13.70	13.35 ± 5.55	1.23 ± 0.36
**A1**	19.10 ± 16.72	13.75 ± 3.74	1.13 ± 0.44
**A2**	12.45 ± 9.81	13.03 ± 6.78	1.33 ± 0.27
**Normal range**	3–66	4.82–19.5	<1.8

Abbreviations: ACTH = adrenocorticotropic hormone; SD = standard deviation.

**Table 4:** Central tendencies and measures of dispersion of lowest T-score at DXA in the studied groups.

Group	Number (%)	Lowest T-Score Mean ± SD	Lowest T-Score Median (Q1, Q3)	Lowest T-Score Min, Max
**A**	33	−1.45 ± 1.24	−1.40 (−2.30, −0.80)	−4.10, 1.30
**A1**	14	−0.33 ± 0.72	−0.55 (−0.85, 0.15)	−1.00, 1.30
**A2**	19	−2.27 ± 0.81	−2.10 (−2.70, −1.70)	−4.10, −1.10
**B**	33	−1.37 ± 0.99	−1.40 (−2.20, −0.80)	−3.60, 1.00
**B1**	14	−0.46 ± 0.56	−0.45 (−1.00, −0.20)	−1.00, 1.00
**B2**	19	−2.04 ± 0.63	−2.00 (−2.50, −1.45)	−3.60, −1.20
***p*-value between:**				
**Groups A and B**			0.785	
**Groups A1 and A2**			**<0.001**	
**Groups A1 and B1**			0.580	
**Groups A2 and B2**			0.334	
**Groups B1 and B2**			**<0.001**	

Abbreviations: SD = standard deviation, Q = quartile, min = minimum, max = maximum. Bold means statistical significant

**Table 5:** BMD and T-score at lumbar, femoral neck, and total hip sites in the studied groups (equality of means test between group A and B, A1 and A2, A1 and B1, A2 and B2, respectively, and B1 and B2).

Group	Lumbar BMD (g/cm^2^) Mean ± SD	Lumbar T-Score Mean ± SD	Femoral Neck BMD (g/cm^2^) Mean ± SD	Femoral Neck T-Score Mean ± SD	Total Hip BMD (g/cm^2^) Mean ± SD	Total Hip T-Score Mean ± SD
**A**	1.038 ± 0.182	−1.09 ± 1.54	0.900 ± 0.147	−0.95 ± 1.13	0.981 ± 0.178	−0.18 ± 1.44
**A1**	1.177 ± 0.139	0.18 ± 1.03	1.018 ± 0.101	0.02 ± 0.78	1.101 ± 0.099	0.79 ± 0.82
**A2**	0.935 ± 0.138	−2.03 ± 1.12	0.828 ± 0.123	−1.54 ± 0.88	0.834 ± 0.138	−1.37 ± 1.10
**B**	1.059 ± 0.162	−1.00 ± 1.34	0.900 ± 0.142	−1.00 ± 1.02	0.988 ± 0.130	−0.14 ± 1.04
**B1**	1.208 ± 0.119	0.24 ± 0.98	1.012 ± 0.108	−0.19 ± 0.78	1.098 ± 0.099	0.71 ± 0.78
**B2**	0.949 ± 0.080	−1.91 ± 0.67	0.817 ± 0.102	−1.59 ± 0.73	0.906 ± 0.081	−0.77 ± 0.70
***p*-value between:**						
**Groups A and B**	0.621	0.799	0.997	0.859	0.886	0.920
**Groups A1 and A2**	**<0.001**	**<0.001**	**<0.001**	**<0.001**	**<0.001**	**<0.001**
**Groups A1 and B1**	0.269	0.441	0.450	0.254	0.470	0.407
**Groups A2 and B2**	0.699	0.702	0.766	0.850	0.092	0.095
**Groups B1 and B2**	**<0.001**	**<0.001**	**<0.001**	**<0.001**	**<0.001**	**<0.001**

Abbreviations: BMD = bone mineral density; SD = standard deviation. Bold means statistical significant.

**Table 6:** Analysis of 10-year probability of major osteoporotic fractures and hip fracture according to the current risk estimator (conventional) FRAX model.

Group	FRAX1 (%) Median (Q1, Q3)	FRAX2 (%) Median (Q1, Q3)	FRAX3 (%) Median (Q1, Q3)	FRAX4 (%) Median (Q1, Q3)
**A**	4.00 (2.00, 6.55)	3.50 (1.85, 5.75)	0.70 (0.20, 2.05)	0.45 (0.15, 1.40)
**A1**	2.20 (1.40, 4.10)	1.85 (1.40, 3.10)	0.30 (0.10, 0.80)	0.15 (0.00, 0.20)
**A2**	4.70 (3.35, 7.15)	4.35 (3.20, 6.20)	0.90 (0.45, 2.70)	0.75 (0.30, 1.90)
**B**	3.70 (2.70, 5.30)	3.72 (2.79, 4.64)	0.50 (0.40, 1.20)	0.40 (0.17, 0.85)
**B1**	3.95 (3.10, 5.30)	2.95 (2.61, 4.00)	0.65 (0.40, 1.20)	0.17 (0.10, 0.40)
**B2**	3.50 (2.55, 5.00)	4.27 (2.90, 5.63)	0.50 (0.30, 1.25)	0.65 (0.32, 1.60)
***p*-value between:**				
**Groups A and B**	0.932	0.510	0.948	>0.999
**Groups A1 and A2**	**0.050**	**0.004**	**0.037**	**0.002**
**Groups A1 and B1**	0.089	**0.030**	0.083	0.316
**Groups A2 and B2**	0.212	0.888	0.191	0.815
**Groups B1 and B2**	0.653	**0.037**	0.700	**0.002**

Abbreviations: Q = quartile; FRAX1 = 10-year probability of major osteoporotic fractures calculated without femoral neck BMD; FRAX2 = 10-year probability of major osteoporotic fractures calculated with femoral neck BMD; FRAX3 = 10-year probability of hip fracture calculated without femoral neck BMD; FRAX4 = 10-year probability of hip fracture calculated with femoral neck BMD. Bold means statistical significant.

**Table 7:** Analysis of 10-year probability of major osteoporotic fractures and hip fracture according to the novel risk estimator FRAXplus model (* green font within the table means FRAXplus model for major osteoporotic fractures risk estimation; ** bluefont within the table means FRAXplus model for hip fracture risk estimation).

Group	FRAXplus1 (%) * Median (Q1, Q3)	FRAXplus2 (%) ** Median (Q1, Q3)
**A**	2.80 (1.55, 4.40)	0.30 (0.10, 1.10)
**A1**	1.55 (1.30, 2.50)	0.10 (0.00, 0.10)
**A2**	3.30 (2.70, 4.60)	0.55 (0.30, 1.40)
**B**	3.00 (2.25, 3.45)	0.30 (0.10, 0.65)
**B1**	2.65 (2.30, 3.20)	0.10 (0.10, 0.30)
**B2**	3.25 (2.20, 4.30)	0.45 (0.30, 1.20)
***p*-value between:**		
**Groups A and B**	0.411	0.707
**Groups A1 and A2**	**0.004**	**<0.001**
**Groups A1 and B1**	**0.013**	0.064
**Groups A2 and B2**	0.864	0.767
**Groups B1 and B2**	0.125	**0.002**

Abbreviations: Q = quartile; FRAXplus1 = 10-year probability of major osteoporotic fractures calculated with lumbar BMD; FRAXplus2 = 10-year probability of hip fracture calculated with lumbar BMD. Bold means statistical significant.

**Table 8:** Analysis of FRAX results versus FRAXplus1 and FRAXplus2 within the studied groups (* green font within the table means FRAXplus model for major osteoporotic fractures risk estimation; ** blue font within the table means FRAXplus model for hip fracture risk estimation).

Group	*p* -Value Between FRAXplus1 and FRAX1 *	*p* -Value Between FRAXplus1 and FRAX2	*p* -Value Between FRAXplus2 and FRAX3 **	*p* -Value Between FRAXplus2 and FRAX4
**A**	**<0.001**	**<0.001**	**0.023**	**<0.001**
**A1**	**0.011**	**0.008**	**0.011**	0.059
**A2**	**0.009**	**<0.001**	0.191	**<0.001**
**B**	**<0.001**	**<0.001**	**0.004**	**<0.001**
**B1**	**<0.001**	**0.002**	**<0.001**	**0.022**
**B2**	**0.031**	**<0.001**	0.483	**<0.001**

Abbreviations: FRAX1 = 10-year probability of major osteoporotic fractures calculated without femoral neck BMD; FRAX2 = 10-year probability of major osteoporotic fractures calculated with femoral neck BMD; FRAX3 = 10-year probability of hip fracture calculated without femoral neck BMD; FRAX4 = 10-year probability of hip fracture calculated with femoral neck BMD; FRAXplus1 = 10-year probability of major osteoporotic fractures calculated with lumbar BMD; FRAXplus2 = 10-year probability of hip fracture calculated with lumbar BMD. Bold means statistical significant.

**Table 9:** Correlation coefficients between FRAXplus1 and FRAXplus2, and age, years since menopause, BMI, lumbar BMD, femoral neck BMD, and total hip BMD in the studied groups. (* green font within the table means FRAXplus model for major osteoporotic fractures risk estimation; ** blue font within the table means FRAXplus model for hip fracture risk estimation).

FRAXplus1 *	Age (Years)	Years Since Menopause	BMI (kg/m^2^)	Lumbar BMD (g/cm^2^)	Femoral Neck BMD (g/cm^2^)	Total Hip BMD (g/cm^2^)
**A**	r = 0.764 ***p* < 0.001**	r = 0.614 ***p* < 0.001**	r = −0.170 *p* = 0.212	r = −0.373 ***p* = 0.006**	r = −0.581 ***p* < 0.001**	r = −0.563 ***p* = 0.002**
**A1**	r = 0.874 ***p* < 0.001**	r = 0.730 ***p* = 0.005**	r = −0.114 *p* = 0.652	r = −0.0.45 *p* = 0.857	r = −0.270 *p* = 0.281	r = −0.473 *p* = 0.105
**A2**	r = 0.669 ***p* < 0.001**	r = 0.540 ***p* = 0.002**	r = −0.047 *p* = 0.790	r = −0.251 *p* = 0.149	r = −0.667 ***p* < 0.001**	r = −0.444 *p* = 0.095
**B**	r = 0.614 ***p* < 0.001**	r = 0.351 ***p* = 0.006**	r = −0.167 *p* = 0.201	r = −0.309 ***p* = 0.014**	r = −0.480 ***p* < 0.001**	r = −0.415 ***p* < 0.001**
**B1**	r = 0.590 ***p* = 0.005**	r = 0.316 *p* = 0.123	r = −0.356 *p* = 0.086	r = −0.078 *p* = 0.701	r = −0.256 *p* = 0.207	r = −0.189 *p* = 0.351
**B2**	r = 0.708 ***p* < 0.001**	r = 0.445 ***p* = 0.013**	r = −0.120 *p* = 0.511	r = −0.348 ***p* = 0.048**	r = −0.870 ***p* < 0.001**	r = −0.616 ***p* < 0.001**
FRAXplus2 **	**Age (years)**	**Years since menopause**	**BMI**	**Lumbar BMD (g/cm^2^)**	**Femoral neck BMD** **(g/cm^2^)**	**Total hip BMD (g/cm^2^)**
**A**	r = 0.606 ***p* < 0.001**	r = 0.555 ***p* < 0.001**	r = −0.227 *p* = 0.108	r = −0.476 ***p* < 0.001**	r = −0.795 ***p* < 0.001**	r = −0.807 ***p* < 0.001**
**A1**	r = 0.485 *p* = 0.076	r = 0.414 *p* = 0.136	r = −0.267 *p* = 0.327	r = −0.211 *p* = 0.434	r = −0.580 ***p* = 0.032**	r = −0.737 ***p* = 0.020**
**A2**	r = 0.569 ***p* = 0.001**	r = 0.553 ***p* = 0.002**	r = −0.082 *p* = 0.645	r = −0.223 *p* = 0.207	r = −0.737 ***p* < 0.001**	r = −0.592 ***p* = 0.028**
**B**	r = 0.403 ***p* = 0.002**	r = 0.263 ***p* = 0.047**	r = −0.108 *p* = 0.424	r = −0.463 ***p* < 0.001**	r = −0.802 ***p* < 0.001**	r = −0.644 ***p* < 0.001**
**B1**	r = 0.260 *p* = 0.259	r = 0.127 *p* = 0.575	r = −0.273 *p* = 0.234	r = −0.097 *p* = 0.664	r = −0.736 ***p* = 0.001**	r = −0.430 ***p* = 0.054**
**B2**	r = 0.563 ***p* = 0.002**	r = 0.423 ***p* = 0.018**	r = −0.211 *p* = 0.247	r = −0.348 ***p* = 0.048**	r = −0.870 ***p* < 0.001**	r = −0.616 ***p* < 0.001**

Abbreviations: BMI = body mass index; BMD = bone mineral density; FRAXplus1 = 10-year probability of major osteoporotic fractures calculated with lumbar BMD; FRAXplus2 = 10-year probability of hip fracture calculated with lumbar BMD. Bold means statistical significant.

## Data Availability

All the available data are already within the paper.

## Abbreviations

The following abbreviations are used in this manuscript:

ACTH	Adrenocorticotropic Hormone
BMI	body mass index
BMD	bone mineral density
DXA	Dual-Energy X-ray Absorptiometry
FRAX	Fracture Risk Assessment Tool
FRAX1	10-year probability of major osteoporotic fractures calculated without femoral neck bone mineral density
FRAX2	10-year probability of major osteoporotic fractures calculated with femoral neck bone mineral density
FRAX3	10-year probability of hip fracture calculated without femoral neck bone mineral density
FRAX4	10-year probability of hip fracture calculated with femoral neck BMD
FRAXplus1	10-year probability of major osteoporotic fractures calculated with lumbar bone mineral density
FRAXplus2	10-year probability of hip fracture calculated with lumbar bone mineral density
MACS	mild autonomous cortisol secretion
SD	standard deviation
Q	quartiles
